# Resveratrol modulates pancreatic proteotoxic stress and metabolic dysfunction in human IAPP transgenic mice

**DOI:** 10.3389/fendo.2026.1871780

**Published:** 2026-08-14

**Authors:** Sarai Iglesias-Fortes, Ana García-Aguilar, Gema García, Carmen Rodríguez-Cueto, Javier Fernández-Ruiz, Olmo Martín-Cámara, J. Carlos Menéndez, Manuel Benito, Carlos Guillén

**Affiliations:** 1 Departamento de Bioquímica y Biología Molecular, Facultad de Farmacia, Universidad Complutense de Madrid, Madrid, Spain; 2 Departamento de Bioquímica y Biología Molecular, Facultad de Farmacia, Universidad Complutense de Madrid, Fundación para la Investigación Biomédica del Hospital Clínico San Carlos (IdISSC), Madrid, Spain; 3 Department of Pharmacology, Pharmacognosy and Botany, Faculty of Pharmacy, Complutense University of Madrid, Madrid, Spain; 4 Centro de Investigación Biomédica en Red de Diabetes y Enfermedades Metabólicas Asociadas (CIBERDEM), Instituto de Salud Carlos III, Madrid, Spain; 5 Instituto de Investigaciones Biomédicas Sols-Monreale, Consejo Superior de Investigaciones Científicas/Universidad Autónoma de Madrid, Madrid, Spain; 6 Instituto Universitario de Investigación en Neuroquímica, Departamento de Bioquímica y Biología Molecular, Facultad de Medicina, Universidad Complutense, Madrid, Spain; 7 Centro de Investigación Biomédica en Red de Enfermedades Neurodegenerativas (CIBERNED), Madrid, Spain; 8 Instituto Ramón y Cajal de Investigación Sanitaria (IRYCIS), Madrid, Spain; 9 Unidad de Química Orgánica y Farmacéutica, Departamento de Química en Ciencias Farmacéuticas, Facultad de Farmacia, Universidad Complutense, Madrid, Spain

**Keywords:** amylin aggregates, brain, liver, mitochondrial dynamics, pancreas, resveratrol, type 2 diabetes

## Abstract

Amylin is a peptide hormone co-secreted with insulin by pancreatic β-cells that regulates glucose homeostasis and satiety. In humans, amylin exhibits a strong propensity to aggregate, contributing to β-cell dysfunction and the development of type 2 diabetes mellitus (T2D). Emerging evidence suggests that amylin aggregates may exert systemic effects beyond the pancreas, potentially affecting peripheral tissues and the brain. However, *in vivo* evidence supporting this multi-organ involvement remains limited. In this study, we investigated the impact of human amylin aggregation across metabolic tissues using a transgenic mouse model expressing human IAPP (TghIAPP), fed either a standard diet (STD) or a high-fat diet (HFD), and evaluated the therapeutic potential of resveratrol. HFD-fed TghIAPP mice developed impaired glucose tolerance, increased amyloid aggregation, and enhanced markers of apoptosis and senescence in pancreatic islets. These alterations were associated with activation of endoplasmic reticulum (ER) stress pathways. Resveratrol treatment significantly reduced pancreatic amyloid burden, attenuated ER stress signaling, and improved markers of β-cell integrity. In the liver, TghIAPP mice exhibited increased lipid accumulation, inflammatory markers, and amyloid oligomer deposition, although the effects of resveratrol were modest and did not consistently reach statistical significance. In the brain, amyloid aggregates were detected in the hippocampus; however, resveratrol-induced changes in aggregate load and behavioral performance were limited and should be interpreted cautiously. Overall, these findings support a robust role for amylin aggregation in pancreatic dysfunction and highlight resveratrol as a modulator of pancreatic proteotoxic stress. The observed alterations in liver and brain suggest potential systemic effects of amylin aggregates that warrant further investigation.

## 1 Introduction

Type 2 diabetes mellitus (T2D) is a complex metabolic disorder characterized by insulin resistance and progressive pancreatic β-cell dysfunction. A key pathological feature of T2D is the aggregation of islet amyloid polypeptide (IAPP, also known as amylin), a peptide hormone co-secreted with insulin by β-cells. In humans, amylin has a strong intrinsic tendency to misfold and form toxic oligomers and fibrils, which accumulate within pancreatic islets and contribute to β-cell failure (1–3). The cytotoxic effects of amylin aggregates have been widely attributed to the induction of endoplasmic reticulum (ER) stress, mitochondrial dysfunction, oxidative stress, and apoptosis (4–6). These processes ultimately impair β-cell viability and insulin secretion, reinforcing the central role of amylin proteotoxicity in the progression of T2D. While the pancreatic effects of amylin aggregation are well established, increasing evidence suggests that misfolded amylin species may also exert systemic effects beyond the pancreas (7). In this context, circulating amylin aggregates and oligomers have been detected in peripheral tissues and have been proposed to contribute to metabolic dysfunction in organs such as the liver (8, 9). In addition, recent studies have suggested a possible link between amylin aggregation and neurodegenerative processes, including the presence of amylin deposits in the brain and their potential interaction with other amyloidogenic proteins (10–12). However, the *in vivo* extent, tissue specificity, and functional relevance of these extra-pancreatic effects remain incompletely understood.

Natural polyphenols have attracted considerable attention as potential modulators of protein aggregation. Among them, resveratrol has been shown to interfere with amyloid formation and reduce proteotoxic stress in multiple experimental systems (13–15). Proposed mechanisms include stabilization of non-toxic protein conformations, inhibition of oligomer formation, and modulation of cellular stress-response pathways such as ER stress and autophagy (14–16). Despite these promising properties, the *in vivo* effects of resveratrol on amylin aggregation and its potential impact across different tissues have not been fully characterized.

In the present study, we used a transgenic mouse model expressing human IAPP (TghIAPP) to investigate the tissue-specific consequences of amylin aggregation under metabolic stress conditions induced by a high-fat diet. We further evaluated the effects of resveratrol on amyloid deposition, cellular stress pathways, and functional outcomes in pancreatic, hepatic, and brain tissues. By combining metabolic, histological, and molecular analyses, this study aims to clarify the relative contribution of amylin aggregation across tissues and to assess the potential of resveratrol as a modulator of amylin-induced proteotoxicity *in vivo*.

## 2 Material and methods

### 2.1 Mice model

In this study, a mouse model expressing human amylin in the pancreas was used, since endogenous murine amylin is non-amyloidogenic and does not form aggregates. The hemizygous TghIAPP mouse strain, developed by Jackson Laboratories^®^ (strain #008232) on an FVB/N genetic background, was crossbred with wild-type (Wt) C57BL/6 mice to maintain heterozygosity of the human islet amyloid polypeptide (hIAPP) gene. Consequently, all animals used in this study had a mixed C57BL/6-FVB/N genetic background. These Tg-hIAPP mice have already been used in previous studies (7), and hybrid mice additionally provide an improved model for neurobehavioral studies (17). As described by Jackson Laboratories, this transgenic mouse model expresses human amylin (hIAPP) driven by the rat insulin II promoter. Although hIAPP mRNA is present in the pancreas, kidney, and stomach, the corresponding protein has been detected solely in pancreatic tissue.

The experimental design included the following experimental groups: Wt STD Vehicle (n = 5), Wt STD RESV (n = 6), Wt HFD Vehicle (n = 7), Wt HFD RESV (n = 7), Tg STD Vehicle (n = 8), Tg STD RESV (n = 8), Tg HFD Vehicle (n = 7), and Tg HFD RESV (n = 10). The corresponding sample size is also indicated in each figure legend.

The mice were weaned at one month of age and subsequently maintained on either a standard diet (STD) or a high-fat diet (HFD) until 8 months of age. In the final three weeks prior to euthanasia, mice were administered daily by intraperitoneal (IP) injections of either a vehicle solution (2% ethanol v/v in phosphate-buffered saline) or resveratrol at a dose of 40 mg/kg, depending on the assigned treatment group. Treatment allocation was randomized.

Glucose and insulin tolerance tests (GTT and ITT, respectively) were performed following the treatment period. All animal procedures were conducted in accordance with internationally accepted standards for animal care and use and were approved by the Institutional Animal Care and Use Committee (protocol number PROEX 112.2/21). Experimental methods were reported in compliance with the ARRIVE (Animal Research: Reporting of *In Vivo* Experiments) guidelines. In order to obtain rapidly the samples and, especially the brain, the animals were euthanized by rapid decapitation.

### 2.2 Antibodies and reagents

ULK1 (#8054), p-ULK1 (ser757) (#1402), p70 (#9202), p-p70 (#9205), PERK (#3192), p-PERK (#3179), eif2α (#5324), p-eif2α (#9721), Bip (#3177), DRP1(611112), p-DRP1 (#3455), p-S6 (#2211), cleaved Caspase-3 (#9661) and Ki67 (8D5, #9449) were from Cell Signalling Technology. Mfn2 (ab56889) A11(AHB0052), β-actin (A5316) were from Abcam. Chop (sc-793), p21 (sc-397), caspase-1 (sc-56036), Mfn1 (sc-50330) and S6 (sc-74459) were from Santa Cruz Technology. Horseradish peroxidase (HRP)-conjugated secondary antibodies were obtained from GE Lifesciences: anti-rabbit (NA934) and anti-mouse (NA931). Resveratrol (sigma R5010-100MG).

### 2.3 Western blot

Tissue samples were collected and homogenized using a Polytron homogenizer in an ice-cold lysis buffer composed of 5 mM EGTA, 5 mM EDTA,1% (v/v) Nonidet-P40, 50 mM Tris-HCl (pH 7.5), 20 mM NaF, 150 mM NaCl, 2 µg/mL leupeptin, 10 µg/mL aprotinin, 1 mM PMSF, and 1 mM sodium orthovanadate. The resulting homogenates were subsequently sonicated and centrifuged at 13,000 rpm for 15 minutes at 4 °C to remove cellular debris. Protein concentrations were quantified using the Bradford assay with the Bio-Rad^®^ reagent (Hercules, CA, USA), following the manufacturer’s instructions. Equal amounts of total protein from each sample were subjected to SDS-PAGE, and proteins were then transferred onto Immobilon PVDF membranes (Merck Millipore, Burlington, MA, USA) using a standard wet transfer protocol for Western blotting. Following transfer, membranes were blocked with 5% bovine serum albumin (Albumin-BSA-Fraction V for Western blotting, Panreac^®^) and incubated overnight at 4 °C with primary antibodies. The following day, membranes were washed with TTBS (Tris-buffered saline with Tween 20) and subsequently incubated with secondary antibodies, also diluted in TTBS. After incubation, excess secondary antibody was removed by washing the membranes three times with TTBS. Protein bands were visualized using enhanced chemiluminescence (ECL) detection reagents (GE Healthcare, Little Chalfont, UK), following the manufacturer’s Western blotting protocol and images were taken using the ChemiDoc™ XRS+ System (Bio-Rad^®^).

### 2.4 Immunohistochemistry

Paraffin-embedded tissue sections were initially deparaffinized using a commercial xylene substitute, followed by gradual rehydration through a graded series of ethanol solutions and subsequent rinsing in tap and distilled water. Antigen retrieval was then performed by heating the sections in a 10 mM sodium citrate buffer (pH 6.0) to reverse cross-linking induced by aldehyde fixation. Endogenous peroxidase activity was quenched by incubation with 3% hydrogen peroxide. To minimize non-specific antibody binding, sections were incubated with a blocking solution consisting of 10% goat serum in PBT. Thereafter, samples were incubated with the primary antibody overnight at 4 °C. On the following day, sections were washed and subsequently incubated with an HRP-conjugated secondary antibody. Signal detection was achieved using the 3,3’-diaminobenzidine (DAB) chromogenic substrate, which yields a brown precipitate upon oxidation in the presence of H_2_O_2_ and horseradish peroxidase. The enzymatic reaction was terminated after 10 minutes by immersion in distilled water, followed by counterstaining with hematoxylin to visualize cell nuclei. Finally, sections underwent progressive dehydration and were mounted with a permanent mounting medium before being air-dried at room temperature. All images were taken at 40X. Image J 1.45 software (National Institutes of Health, Bethesda, MD, USA) was used. All tissues were sectioned at 10 µm thickness, except the brain, which was sectioned at 30 µm.

### 2.5 Immunofluorescence

Tissues were fixed in 4% paraformaldehyde (PFA) overnight, transferred to 30% sucrose, and kept overnight. Subsequently, tissues were embedded in OCT and sectioned using a cryostat. All tissues were sectioned at 10 µm thickness, except the brain, which was sectioned at 30 µm. The tissue sections were thawed at room temperature for 10 minutes and briefly rinsed with 1× PBS to remove residual OCT. A hydrophobic barrier was then drawn around the tissue sections using a hydrophobic marker to ensure precise application of reagents. Permeabilization was carried out by incubating the sections with 0.3% Triton X-100 in 1X PBS for 15 minutes at room temperature. Following this step, tissues were incubated for 1 hour at room temperature in a blocking solution containing 10% BSA and 0.1% Triton X-100 in PBT, then washed three times with the same buffer. To visualize amyloid fibrils, the sections were incubated with 10 μM Thioflavin T, followed by three additional washes with PBT. Nuclear counterstaining was subsequently performed with DAPI (1 μg/ml) for 10 minutes. The samples were mounted using Fluoromount-G^®^ (SouthernBiotech), and coverslips were carefully applied to prevent the formation of air bubbles. Mounted preparations were allowed to dry in the dark at 4 °C for 24 hours. High-resolution images (1024 × 1024 pixels) were acquired using a Leica SP-8 laser scanning confocal microscope at the Research Support Centre (CAI) for Microscopy and Flow Cytometry, Complutense University of Madrid.

### 2.6 β-galactosidase staining

To assess senescence in various tissues, a β-galactosidase staining kit (Cell Signaling Technology^®^, #9860) was utilized. Tissue sections embedded in OCT were employed for this analysis. Following sample thawing, a brief wash with 1X PBS was performed to remove the OCT, after which the fixation solution provided by the kit was applied, and the samples were incubated at room temperature for 15 minutes. Subsequently, another wash with 1X PBS was carried out, and the β-galactosidase staining solution, prepared by combining the 1X of staining solution, solution A, solution B, and X-Gal dissolved in DMSO as per the kit instructions, was applied with the pH adjusted to 6.0. The samples were then incubated for 48 hours at 37 °C in a CO2-free incubator to prevent pH changes that could affect the staining process. During incubation, the samples were stored in a humidified chamber to prevent the evaporation of the staining solution and the formation of crystals, which could compromise the preparation. All images were taken at 40X.

### 2.7 Oil Red staining

The Oil-Red-O staining method was employed to visualize and quantify lipid content in tissue samples using the diazo dye Oil-Red O. To prepare the working solution, 0.36 g of commercial Oil-Red (Sigma-Aldrich^®^, O0625) was dissolved in 120 mL of 2-propanol, then diluted with water in a 3:2 ratio and allowed to rest for 10 minutes before filtration. For staining, OCT-embedded tissue sections were thawed and fixed in 4% formaldehyde for 10 minutes. A brief wash with 60% isopropanol was performed, followed by incubation in the filtered Oil-Red solution for 30 minutes. After incubation, tissues were washed again with 60% isopropanol and then with distilled water for 5 minutes. Nuclear staining was performed using Mayer’s hematoxylin for 3 minutes, followed by another wash in distilled water. Finally, the slides were mounted with Aquatex^®^ (Sigma-Aldrich^®^, 1.08562) and left to dry at room temperature. All images were taken at 40X.

### 2.8 Glucose tolerance test and insulin tolerance test

The procedure was conducted following an overnight fasting period of the rodents, and a small blood sample was collected to assess basal glucose levels using an Accu-Chek^®^ Aviva glucometer (Roche). Subsequently, an intraperitoneal injection of glucose was administered at a dose of 2 g per kg of body weight. The initial glucose measurement was recorded as time zero, with subsequent measurements taken at 30-, 60-, 90-, and 120-minutes post-glucose administration.

The insulin tolerance test was performed in ad libitum-fed mice. Animals received an intraperitoneal injection of human insulin (Novo Nordisk^®^) at a dose of 1 U/kg body weight. Blood glucose levels were measured at 15, 30, and 60 minutes after insulin administration. GTT and ITT were statistically analyzed using the area under the curve (AUC) calculated for each individual animal.

### 2.9 Novel Object Recognition test

Experiments were conducted during the animals’ light cycle in a matte black wooden arena (1×1×1 m), with activity recorded via a computer-connected camera. To minimize stress due to environmental changes, experimental cages were placed in the testing room at least 30 minutes before testing for acclimatization. Over the first two days, mice were allowed to explore the empty arena for 10 minutes to familiarize themselves with the environment. On the third day, during the training phase, two identical plastic pipette tip boxes were introduced, and mice explored them for 10 minutes. On the fourth day (test day), one of the familiar objects was replaced with a novel item, a glass jar, and exploration was again allowed for 10 minutes. Object exploration was defined as any behavior involving direct contact with the object using the forepaws, nose, or whiskers, or positioning the nose within 1 cm of the object. The plastic box served as the familiar object, while the jar was considered novel. The formulas used to calculate each of the analyzed parameters are described: The preference index was calculated as the proportion of time spent exploring the novel object over the total exploration time and expressed as a percentage. The recognition index (RI) was calculated as the proportion of time spent exploring the novel object relative to the total exploration time. Finally, the discrimination index was calculated as the difference between the time spent exploring the novel object and the time spent exploring the familiar object, normalized to the total exploration time and expressed as a percentage.

### 2.10 *In silico* analysis

A MolPort search was first performed to assemble a small chemolibrary of commercially available compounds structurally related with resveratrol. Using resveratrol as the query molecule, we selected 22 compounds showing structural variability while maintaining the Tanimoto index ≥ 0.7. The SMILES strings for these ligands were retrieved and converted into individual SDF files, adding explicit hydrogens and assigning AM1-BCC partial charges with AmberTools (18, 19). These structures were then transformed into PDBQT files with the prepareligands4.py utility contained in the MGLTools suite (20).

A blind docking was then performed with AutoDock VINA4 for all the 23 ligands (resveratrol plus 22 MolPort analogues) into a single protofibril composed of 3 monomers (chains E, F and G) obtained from the 3D structure of the pdb 7YL3. Prior to docking, this structure was minimized using 1000 steepest descent steps followed by 1000 conjugate gradient steps in Chimera. This first docking step was employed to determine the preferential binding regions across the fibrillar assembly. After selecting the preferential binding site, a second stage of docking was performed using AutoDock4.2 (21). The grid box was centered in the center of mass of Gly24 (chain E), Ile26 (chain F) and Gln21(chain G) with a grid dimension of 78, 60, 70 (spacing 0.375 Å) along the x, y, z axes. The resulting poses were ranked by predicted binding energy and visually inspected, and the best binding mode for each ligand was selected, retrieving the associated binding energy.

### 2.11 Image acquisition and quantitative analysis

All experimental procedures, image acquisition, and image quantification were performed in a blinded manner, whereas the final data analysis was carried out unblinded. Quantification of the images was performed using Fiji software. For threshold determination, two representative images were selected under blinded conditions, one with high staining intensity and one with low staining intensity, in order to define a threshold range for signal quantification. After this range was established, all images were quantified using the same threshold settings by applying a macro in Fiji, ensuring uniform processing of all samples.

### 2.12 Blinding

Investigators were blinded during image acquisition, quantitative image analysis, Western blot densitometry, and behavioral assessments by identifying samples exclusively with animal identification numbers, without knowledge of the corresponding experimental group. For histological and immunofluorescence analyses, representative images exhibiting high and low staining intensities were initially used to define a common threshold. Once established, the same threshold was applied to all images using an automated ImageJ macro, allowing objective quantification without knowledge of group allocation. Western blot densitometric analyses were also performed using coded samples identified only by animal numbers. Following completion of all quantitative analyses, the data were decoded and assigned to their respective experimental groups for statistical analysis and interpretation. Therefore, investigators were no longer blinded during data analysis and interpretation.

### 2.13 Statistical analysis

Results are expressed as the ± SEM. To assess the normality of the data, the Shapiro-Wilk test was performed. The analysis was conducted using GraphPad Prism version 8.02. Statistical differences between groups were determined using two-way ANOVA with *post-hoc* Tukey test. Differences were considered statistically significant at p<0.05 (*p < 0.05; ** p < 0.01; ***p<0.001, ****p<0.0001).

## 3 Results

### 3.1 HFD promotes metabolic dysfunction and pancreatic β-cell stress in TghIAPP mice

**Figure 1:**
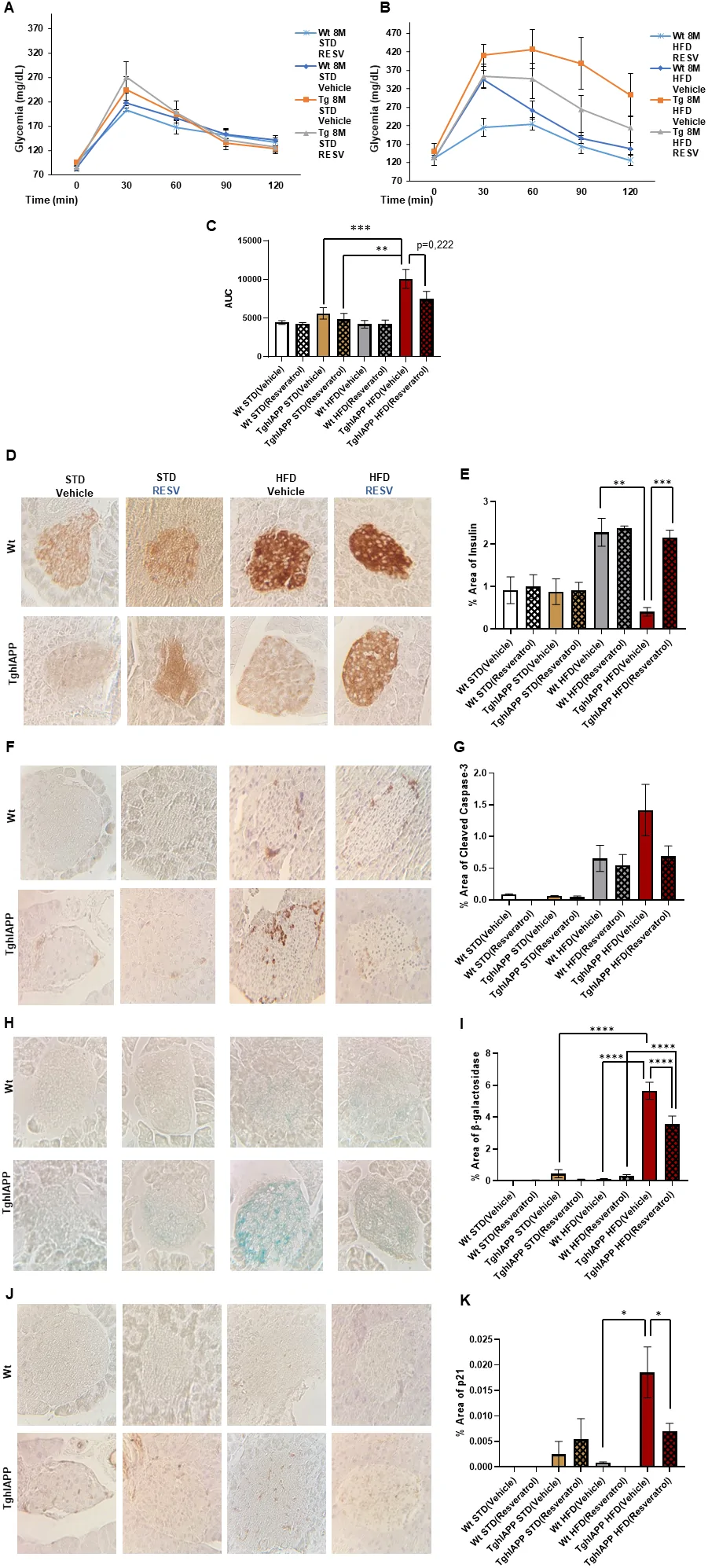
**(A–C)** Glucose tolerance test (GTT) curves for TghIAPP and wild-type (Wt) mice fed either a standard diet (STD, **A**) or high-fat diet (HFD, **B**), and treated with vehicle or resveratrol as appropriate, along with quantification of the area under the curve under HFD (AUC, **C**). **(D, E)** Representative immunohistochemistry images of insulin staining in pancreatic tissue, with corresponding quantification. **(F, G)** Immunohistochemical analysis of cleaved caspase-3 and its quantification on pancreas tissue. **(H, I)** β-galactosidase staining with quantification. **(J, K)** Immunohistochemistry of p21 and corresponding quantification. All data are presented as mean ± standard error of the mean (SEM) (Wt STD Vehicle: n = 5; Wt STD RESV: n = 6; Wt HFD Vehicle: n = 7; Wt HFD RESV: n = 7; Tg STD Vehicle: n = 8; Tg STD RESV: n = 8; Tg HFD Vehicle: n = 7; Tg HFD RESV: n = 10). A two-way ANOVA was performed, with statistical significance defined as follows: *p < 0.05; **p < 0.01; ***p < 0.001; ****p < 0.0001.

TghIAPP mice maintained on HFD exhibited a significant impairment in glucose tolerance relative to their Wt counterparts (**Figures 1B, C**) as well as an insulin resistance state (**Supplementary Figure 1**, lower graph). However, no differences neither in glucose tolerance (**Figure 1A**) nor insulin tolerance (**Supplementary Figure 1**, upper graph) were observed between the groups under STD conditions. Notably, resveratrol administration tended to enhance glucose tolerance, but this effect was confined to TghIAPP mice fed an HFD (**Figure 1C**).

To further investigate pancreatic β-cell integrity, immunohistochemical analysis for insulin was conducted. Results revealed that Wt mice on HFD displayed elevated insulin levels within pancreatic islets compared to those on STD. In contrast, HFD-fed TghIAPP mice treated with vehicle exhibited significantly reduced islet insulin content, not only relative to their Wt counterparts, but also compared to STD-fed mice. Resveratrol treatment was associated with increased islet insulin immunoreactivity in HFD-fed TghIAPP mice to those observed in HFD-fed Wt mice (**Figures 1D, E**).

Given the marked reduction in insulin expression in the islets of HFD-fed TghIAPP mice, the expression of cleaved-caspase-3, a marker of apoptosis, was assessed. These mice displayed increased cleaved caspase-3 protein levels, which were attenuated upon resveratrol treatment (**Figures 1F, G**). To further evaluate tissue homeostasis, pancreatic Ki67 immunostaining was also performed. Although no statistically significant differences were observed among the experimental groups, HFD-fed WT mice showed a tendency toward higher Ki67 immunoreactivity, whereas HFD-fed TghIAPP mice exhibited a comparatively lower proliferative response. Resveratrol treatment tended to increase Ki67 staining in HFD-fed TghIAPP mice (**Supplementary Figures 2A, B**).

In parallel, HFD-fed TghIAPP mice demonstrated a significant increase in cellular senescence within pancreatic islets, as evidenced by elevated β-galactosidase staining and upregulation of the cyclin-dependent kinase inhibitor p21. Importantly, resveratrol administration significantly reduced the expression of both senescence markers consistent with attenuation of senescence-associated markers against HFD-induced cellular aging (**Figures 1H–K**).

### 3.2 Resveratrol attenuates pancreatic amyloid burden and modulates proteotoxic stress signaling

**Figure 2:**
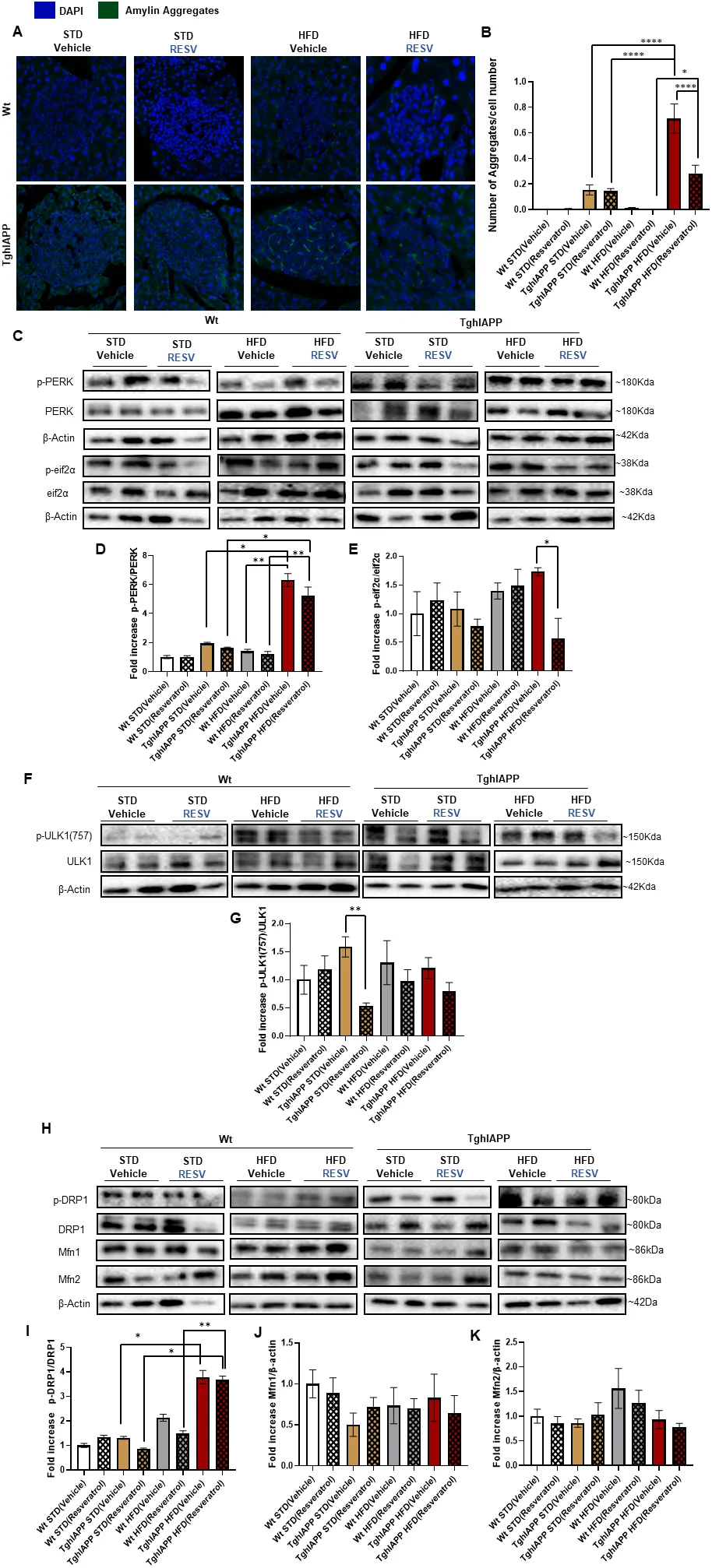
**(A, B)** Representative immunofluorescence images of pancreatic tissue, showing amyloid aggregates labeled with Thioflavin T (green) and nuclei stained with DAPI (blue). Quantification of aggregate number per cell is presented. **(C–E)** Western blot analysis of p- PERK/PERK and p-eIF2α/eIF2α in pancreatic tissue. **(F, G)** p-ULK1 (757)/ULK1 protein levels in pancreatic tissue assessed by Western blot. **(H–K)** Western blot quantification of p-DRP1/DRP1, Mfn1, and Mfn2 protein levels in the pancreas. All data are presented as mean ± standard error of the mean (SEM) (Wt STD Vehicle: n = 5; Wt STD RESV: n = 6; Wt HFD Vehicle: n = 7; Wt HFD RESV: n = 7; Tg STD Vehicle: n = 8; Tg STD RESV: n = 8; Tg HFD Vehicle: n = 7; Tg HFD RESV: n = 10). A two-way ANOVA was performed, with statistical significance defined as follows: *p < 0.05; **p < 0.01; ***p < 0.001; ****p < 0.0001 The grouping of blots was cropped from different parts of the same gel and are mark in a black box.

To determine whether the resveratrol mediated improvements in apoptosis and senescence alongside the observed increase in β-cell markers were attributable to a reduction in amyloid aggregation, pancreatic sections were analyzed by immunofluorescence using Thioflavin T staining. Consistent with the characteristics of the model, pancreatic amyloid aggregates were detected exclusively in TghIAPP mice, with a significantly higher burden observed in those fed an HFD compared to their counterparts maintained on STD. Notably, resveratrol treatment markedly reduced the presence of amyloid aggregates in HFD-fed TghIAPP mice (**Figures 2A, B**).

Furthermore, HFD-fed TghIAPP mice exhibited elevated levels of phosphorylated protein kinase-like ER kinase (p-PERK) and phosphorylated alpha subunit of translation initiation factor 2 (p-eIF2α), indicative of heightened ER stress relative to Wt controls. Resveratrol administration significantly reduced p-eIF2α expression in TghIAPP mice, suggesting a partial alleviation of ER stress (**Figures 2C–E**).

Given the established interplay between ER stress and the mTORC1 signaling pathway, as well as the critical role of this axis in β-cell function, levels of phosphorylated Unc-51-like Kinase 1 at serine 757 [p-ULK1(Ser757)] were examined. TghIAPP mice maintained on an STD exhibited elevated p-ULK1(Ser757) expression, which persisted in those on an HFD (**Figure 2C**). Resveratrol treatment tended to reduce p-ULK1(Ser757) levels in both dietary conditions, although this effect did not reach statistical significance (**Figures 2F, G**).

Additionally, mitochondrial dynamics, a pathway known to be disrupted in the context of amyloid accumulation, were also assessed. HFD-fed TghIAPP mice displayed significantly increased phosphorylation of dynamin-related protein 1 (p-DRP1), coupled with no significant changes in the expression of Mitofusin 1 (Mfn1) or Mitofusin 2 (Mfn2), when compared to their Wt counterparts (**Figures 2H–K**). However, resveratrol treatment had no discernible impact on these mitochondrial parameters.

### 3.3 TghIAPP mice on HFD show hepatic amylin aggregation accompanied by steatosis, inflammation, and cell death

**Figure 3:**
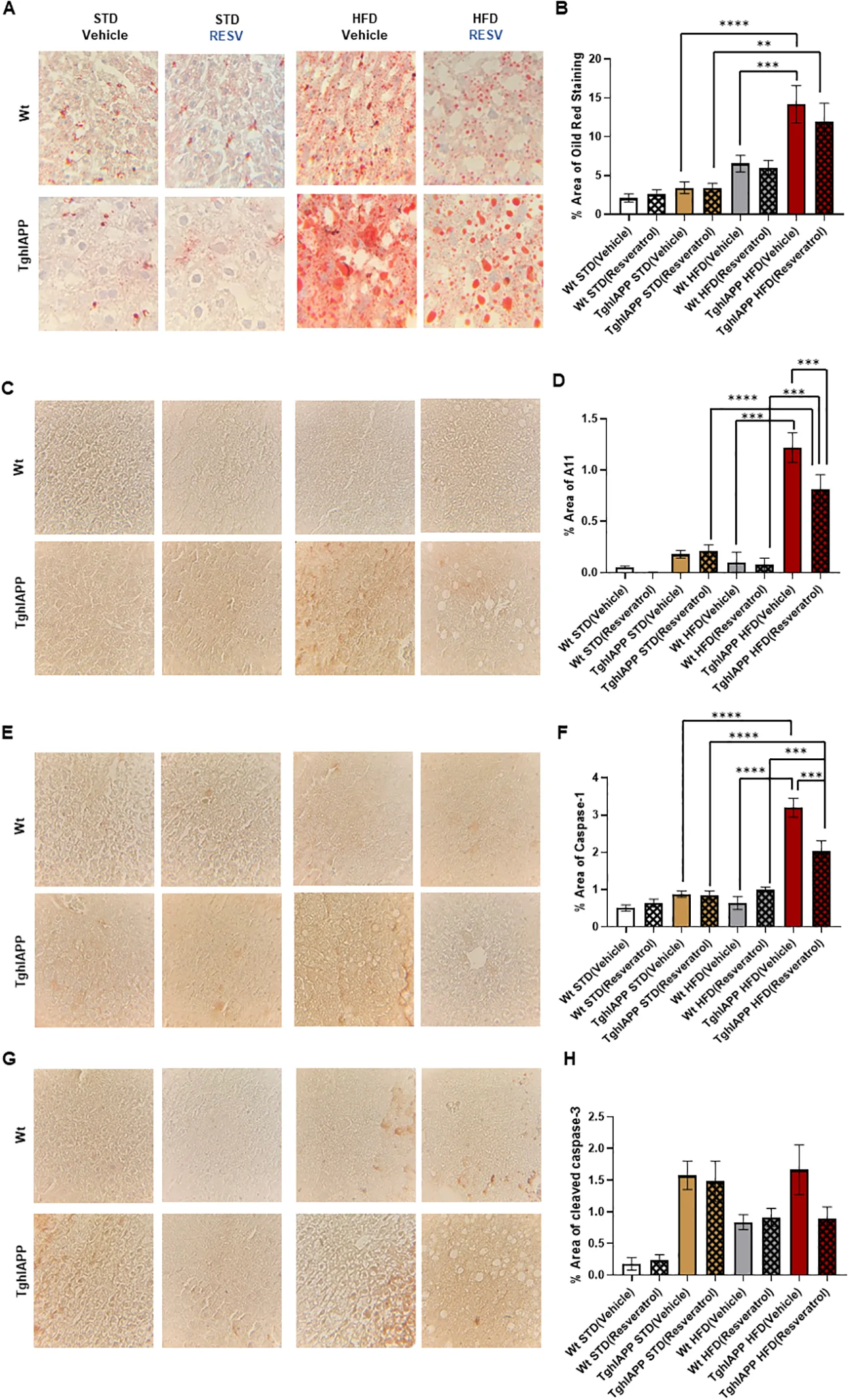
**(A, B)** Oil Red O staining of liver sections, with red signal indicating lipid accumulation; corresponding quantification is provided. **(C, D)** Immunohistochemical analysis of liver sections using the A11 antibody to detect amyloid oligomers, accompanied by quantification. **(E, F)** Immunohistochemistry of liver tissue labeled with caspase-1. **(G, H)** Immunohistochemical staining of cleaved caspase-3 in liver sections, along with quantification. All data are presented as mean ± standard error of the mean (SEM) (Wt STD Vehicle: n = 5; Wt STD RESV: n = 6; Wt HFD Vehicle: n = 7; Wt HFD RESV: n = 7; Tg STD Vehicle: n = 8; Tg STD RESV: n = 8; Tg HFD Vehicle: n = 7; Tg HFD RESV: n = 10). A two-way ANOVA was performed, with statistical significance defined as follows: *p < 0.05; **p < 0.01; ***p < 0.001; ****p < 0.0001.

Given the central role of the liver in the pathophysiology of T2D and the limited evidence in the literature regarding the contribution of amylin to hepatic dysfunction, this study aimed to investigate whether amylin plays a role in liver damage. Initial analyses revealed that TghIAPP mice fed with HFD exhibited a significant increase in hepatic lipid accumulation compared to Wt controls (**Figures 3A, B**). Although resveratrol administration reduced hepatic steatosis in these mice, this effect did not reach statistical significance (**Figure 3B**).

In light of these findings, immunohistochemical analysis was performed using the A11 antibody, which selectively detects amyloid oligomers. Staining was observed exclusively in TghIAPP mice, with a significantly greater accumulation of amyloid oligomers in those fed an HFD (**Figures 3C, D**). Resveratrol treatment tended to diminish the presence of these oligomers, although the reduction did not achieve statistical significance (**Figures 3C, D**).

Moreover, HFD-fed TghIAPP mice demonstrated elevated levels of caspase-1 and cleaved caspase-3 relative to Wt counterparts, indicative of heightened inflammatory and apoptotic activity within the liver (**Figures 3E–H**). Resveratrol treatment was associated with a downward trend in the expression of both proteins; however, this reduction was statistically significant only for caspase-1 (**Figures 3E, F**).

### 3.4 Resveratrol has limited effects on hepatic stress signaling despite modulation of selected mitochondrial markers

**Figure 4:**
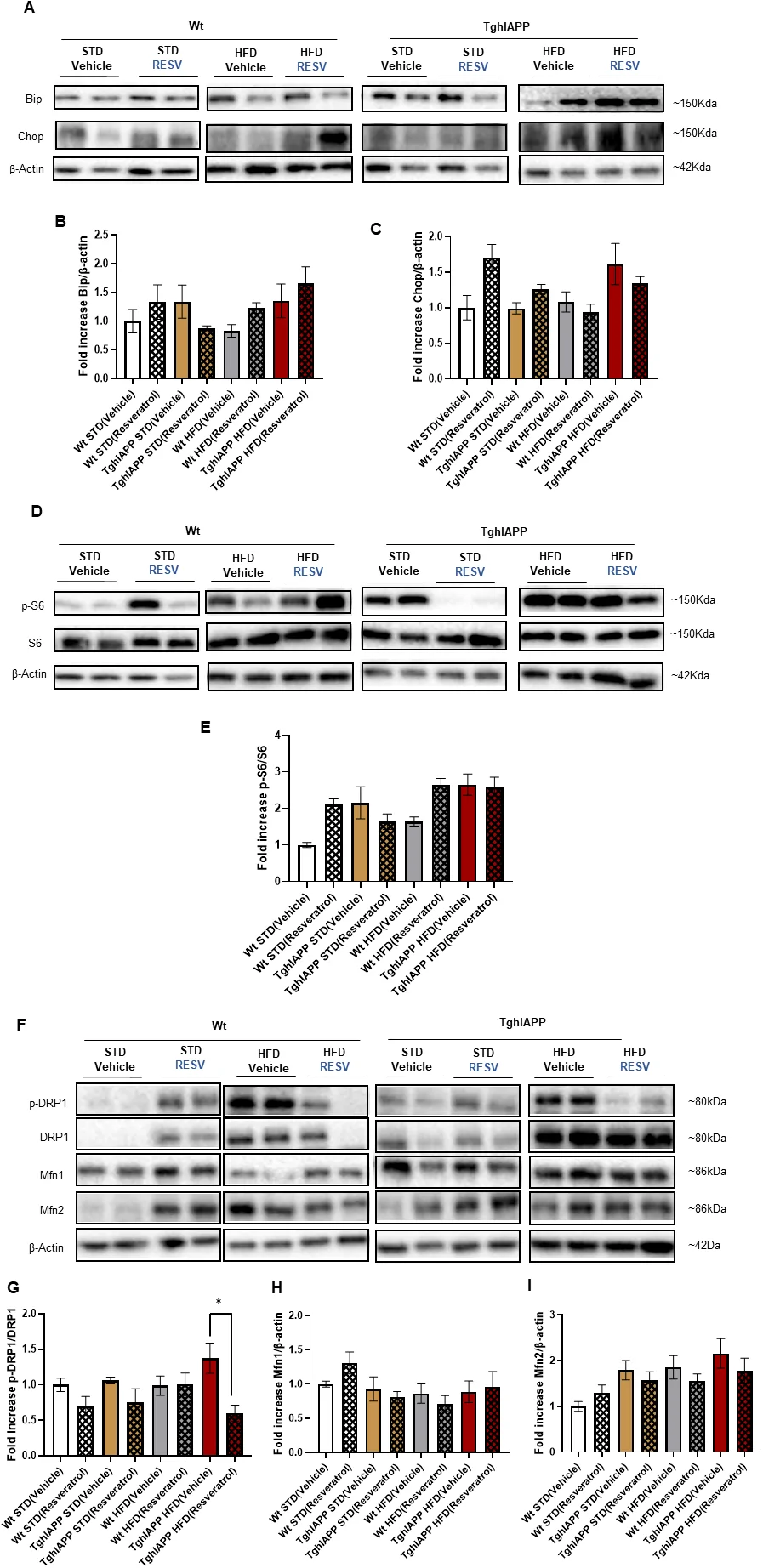
**(A–C)** Protein expression levels of Bip and CHOP in liver tissue assessed by Western blot and quantified. **(D, E)** Western blot analysis of liver tissue showing p-S6/S6 levels, with corresponding quantification. **(F–I)** Western blot analysis of p-DRP1/DRP1, Mfn1, and Mfn2 in liver tissue, with respective quantifications. All data are presented as mean ± standard error of the mean (SEM) (Wt STD Vehicle: n = 5; Wt STD RESV: n = 6; Wt HFD Vehicle: n = 7; Wt HFD RESV: n = 7; Tg STD Vehicle: n = 8; Tg STD RESV: n = 8; Tg HFD Vehicle: n = 7; Tg HFD RESV: n = 10). A two-way ANOVA was performed, with statistical significance defined as follows: *p < 0.05; **p < 0.01; ***p < 0.001; ****p < 0.0001 The grouping of blots was cropped from different parts of the same gel and are mark in a black box.

Given the presence of amyloid oligomers in the liver, downstream cellular pathways known to be affected by protein aggregation were investigated. Compared to Wt controls, TghIAPP mice fed a HFD exhibited elevated expression of the ER stress markers: binding immunoglobulin protein (Bip) and C/EBP homologous protein (Chop), indicating a mild activation of the ER stress response (**Figures 4A–C**). Concurrently, these mice showed enhanced activation of the mTORC1 signaling pathway, as evidenced by increased phosphorylation of ribosomal protein S6 (p-S6), further supporting the notion that amyloid oligomers disrupt hepatic signaling cascades (**Figures 4D, E**). Notably, resveratrol treatment did not modulate these ER stress or mTORC1-related pathways. Additional Ki67 immunostaining revealed a significant increase in HFD-fed Wt mice compared with STD-fed Wt controls, whereas no other statistically significant differences were detected among the experimental groups (**Supplementary Figures 2C, D**).

Additionally, TghIAPP mice on an HFD demonstrated increased levels of p-DRP1, whereas no significant changes were observed in the levels of Mfn1 or Mfn2. Resveratrol also significantly decreased the levels of p-DRP1 (**Figures 4F–I**).

### 3.5 Exploratory hippocampal alterations associated with hIAPP aggregation

The role of amylin in the pathogenesis of neurodegenerative disorders, particularly Alzheimer’s disease, has recently gained increasing scientific attention. In light of this, the present study sought to investigate the hippocampus to determine whether resveratrol exerts any neuroprotective effects in this region. Initial observations revealed the presence of amyloid aggregates in the hippocampus of TghIAPP mice fed a HFD (**Supplementary Figure 3**). These aggregates displayed a non-uniform distribution, with significantly greater accumulation observed in the CA1 subfield and in the dentate gyrus (DG) (**Supplementary Figure 3B**). Although resveratrol treatment did not significantly reduce aggregate burden, a non-significant trend toward decreased accumulation was noted in the DG (**Supplementary Figure 3E**).

**Figure 5:**
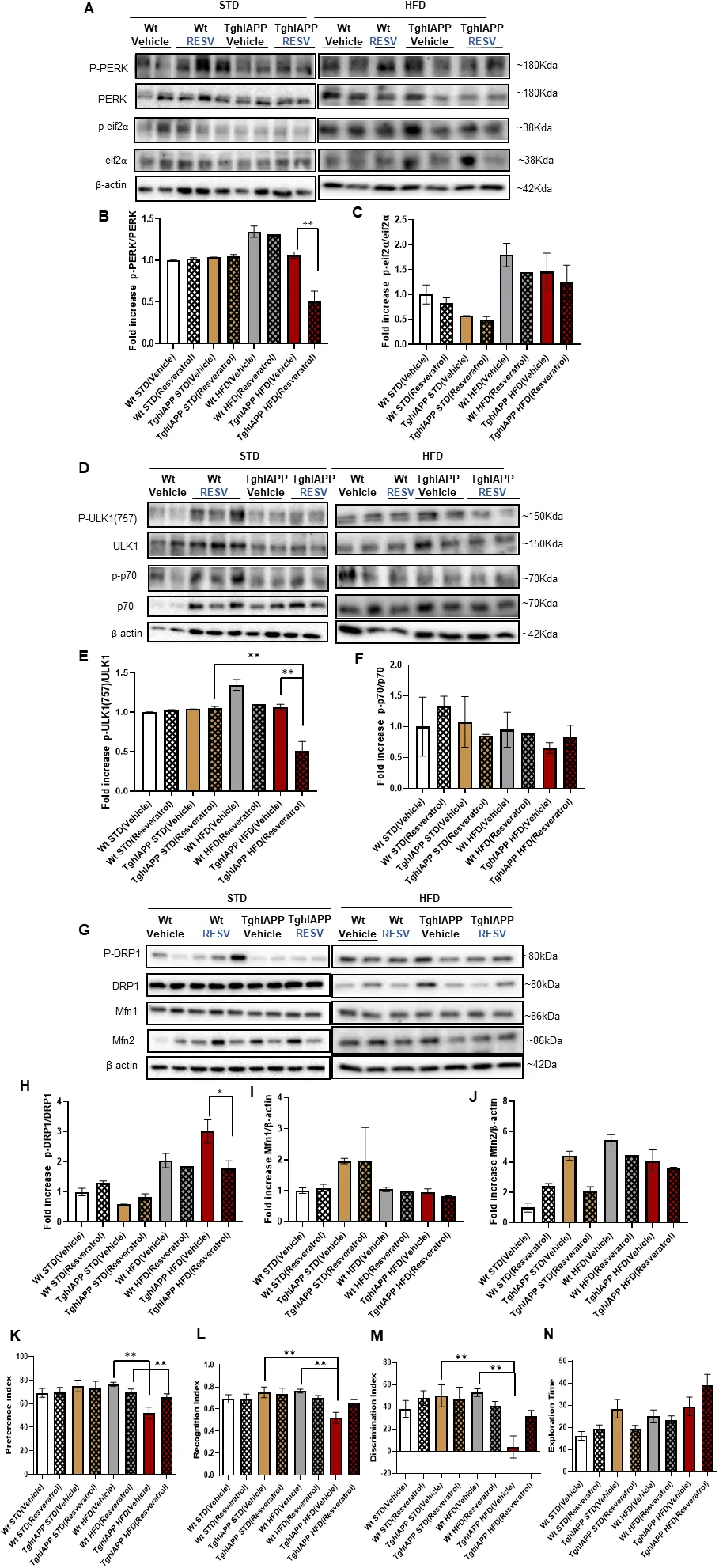
Western blot analysis was conducted on hippocampal tissue, with corresponding quantifications. **(A–C)** Protein levels of p-PERK/PERK and p-eIF2α/p-eIF2α. **(D, E)** Protein levels of p-ULK1–757 and p-p70. **(F–I)** Protein levels of p-DRP1/DRP1, Mfn1, and Mfn2. **(K–N)** Plots of the mean time in the different parameters analyzed in the NOR test. All data are presented as mean ± standard error of the mean (Wt STD Vehicle: n = 5; Wt STD RESV: n = 6; Wt HFD Vehicle: n = 7; Wt HFD RESV: n = 7; Tg STD Vehicle: n = 8; Tg STD RESV: n = 8; Tg HFD Vehicle: n = 7; Tg HFD RESV: n = 10). A two-way ANOVA was performed, with statistical significance defined as follows: *p < 0.05; **p < 0.01; ***p < 0.001; ****p < 0.0001.

Furthermore, resveratrol administration significantly reduced p-PERK levels in hippocampal tissue from HFD-fed Tg-hIAPP mice, indicating an attenuation of ER stress (**Figure 5A**) and its corresponding quantification (**Figure 5B**). However, this effect was not observed in p-eIF2-α signaling (**Figure 5C**). This effect was accompanied by a significant decrease in p-ULK1 (Ser757) levels (**Figures 5D, E**) but not in phosphorylation status of p70S6K (**Figures 5D, F**).

In contrast, mitochondrial dynamics in the hippocampus was more sensitive to the pathological effects of amylin. TghIAPP mice on STD diet demonstrated reduced levels of p-DRP1 alongside increased expression of Mfn1, compared to their respective Wt controls (**Figures 5G–I**). However, the mitochondrial profile in HFD-fed TghIAPP mice was markedly different, with an increase in p-DRP1 and a reduction in Mfn2 expression relative to Wt mice. Notably, resveratrol significantly decreased p-DRP1 levels in this group, suggesting a modulation of selected mitochondrial dynamics markers (**Figures 5G–J**).

In contrast to the hippocampus, no significant pathological alterations were observed in the prefrontal cortex. HFD-fed TghIAPP mice did not exhibit accumulation of amyloid aggregates or notable disruptions in the examined signaling pathways within cortical tissue (**Supplementary Figures 4**, **5**).

Conversely, the recognition and working memory of the mice were evaluated through the Novel Object Recognition (NOR) behavioral assay. TghIAPP mice subjected to an HFD exhibited a significantly reduced preference and recognition indices. Notably, this group was the only one in which some individuals demonstrated a negative discrimination index. No statistically significant rescue in behavioral performance was observed, although a trend toward improvement was detected (**Figures 5K–N**).

### 3.6 *In silico* analysis of potential compounds with antiaggregating activity

In view of the experimental evidence showing that resveratrol reduces amylin aggregation and proteotoxic stress *in vivo*, we became interested in performing a more detailed study of its interaction with aggregated amylin at the molecular level and in the evaluation of the affinity of structurally related small molecules to the same sites in order to facilitate future efforts aiming at the design of resveratrol analogues with an improved profile or better synthetic accessibility. To this end, we assembled a chemolibrary of commercially available compounds from MolPort, using resveratrol as the query structure, and selected 22 analogues based on structural variability while maintaining a Tanimoto similarity index ≥ 0.7 (**Supplementary Figure 6**).

**Figure 6:**
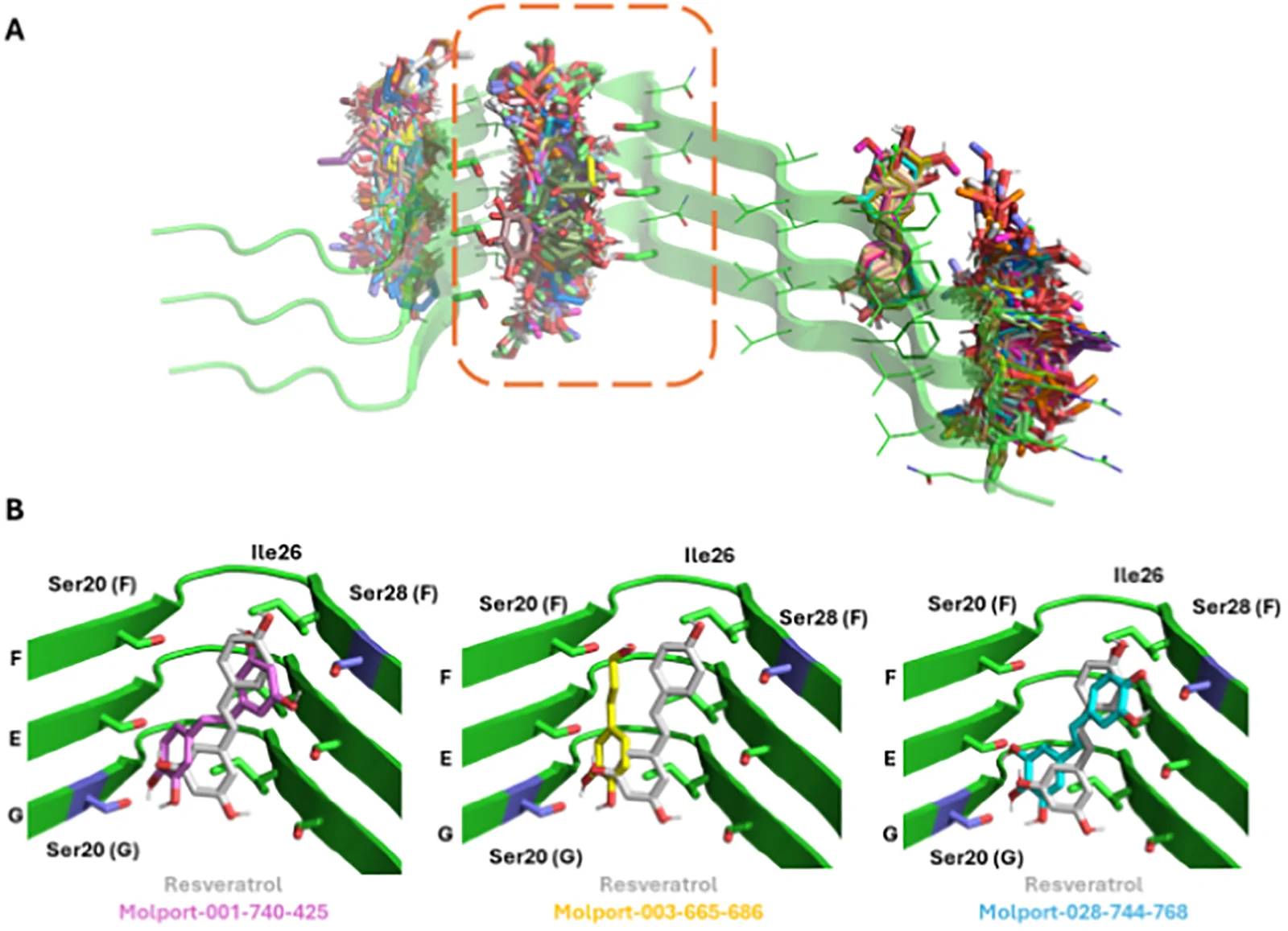
**(A)** Preferential ligand-binding regions identified by blind docking on the amylin protofibril. The selected binding site (residues Ser19- Ser28) is highlighted with an orange dashed box. **(B)** Comparison of the predicted binding poses of the three top ranked compounds (Molport-001-740–425 as pink sticks, Molport-003-665–686 as yellow sticks and Molport-028-744–768 in cyan sticks) with resveratrol (white sticks) docked into the selected binding site of the amylin protofibril (chain **(E–G)** from PDBid: 7YL3, green cartoon).

After ligand and receptor preparation, we performed a two-step docking protocol using an amylin protofibril composed of three aggregated monomers. In the first place, we performed a blind docking using VINA to identify the potential ligand binding sites within the fibrillar assembly. We found four possible binding sites, but 45% of the poses converged on a single binding site, which also showed the most favorable scores (**Figure 6A**). This preferential site corresponds to the conformational kink present in the pdb 7YL3 structure, which includes residues from Ser19 to Ser28, a region which may be implicated in lateral protofibril association and therefore a plausible druggable hotspot for modulating aggregation. Next, we performed a second docking step with AutoDock4.2, focusing in this region. Across the lowest-energy poses of the 23 compounds (**Supplementary Table 1**), ligands consistently formed hydrogen bonds (HB) with Ser20 and Ser23, with residues acting as both, hydrogen bond donors (HBD) and acceptors (HBA), depending on ligand orientation. Ligands which contained multiple phenolic groups frequently established short cooperative hydrogen bond networks, further stabilizing ligand binding into this kink. Also, the presence of aromatic groups enabled CH-π interactions with other non-polar residues such as Ile26 side chain that further improve this binding mode.

Among all the studied compounds, Molport-001-740-425, Molport-003-665–686 and, specially, Molport-028-744–768 showed the most favorable binding energy (**Supplementary Table 1**, **Figure 6B**). These ligands shared a similar binding mode, establishing HB between one or more phenolic groups and Ser20 of chain G in addition to a second HB between hydroxyl groups of the other side of the ligand and Ser28 of chain F or Ser20 (F) in the case of the smaller ligand Molport-003-665-686. It can thus be concluded that ligands with a higher number of phenolic substituents in comparison with resveratrol exhibit increased affinity for this kink, whereas molecules substituted with methoxy or halogen groups show a reduced binding affinity. These results suggest that polyphenolic enrichment enhances ligand affinity for this crucial structural element of the protofibril, identifying several candidates with predicted affinities superior to that of the parent compound.

## 4 Discussion

This study investigates the tissue-specific effects of human amylin aggregation in a transgenic mouse model and evaluates the potential of resveratrol as a modulator of these alterations. Our findings provide robust evidence supporting the role of amylin aggregates in pancreatic dysfunction, while additional observations in liver and brain tissues suggest more moderate and context-dependent effects. Amylin aggregation has been extensively implicated in the pathogenesis of T2D through the induction of β-cell dysfunction and death (3). In line with previous studies, we observed that TghIAPP mice fed a HFD exhibited impaired glucose tolerance, reduced insulin immunoreactivity within pancreatic islets, and increased markers of apoptosis and senescence. These findings reinforce the established association between amyloid deposition and β-cell failure (1, 2). Notably, the absence of significant differences in insulin tolerance suggests that the primary defect in this model lies in insulin secretion rather than peripheral insulin sensitivity, consistent with early stages of T2D progression (22).

Resveratrol treatment significantly reduced pancreatic amyloid burden and attenuated key markers of cellular stress, including ER stress and apoptosis. These findings are consistent with previous reports demonstrating the anti-aggregation properties of resveratrol *in vitro* and *in silico* (14, 23), as well as its protective effects in β-cells exposed to amyloidogenic stress (15). In addition, resveratrol has been shown to modulate cellular senescence in other biological systems, including a reduction in β-galactosidase activity in aging models (24), supporting the observed decrease in senescence markers in pancreatic islets. Mechanistically, resveratrol has been proposed to interfere with amyloid formation by stabilizing non-toxic conformations of IAPP and disrupting its interaction with lipid membranes (14). Our *in-silico* analysis further supports this mechanism by identifying a preferential binding site for resveratrol within the conformational kink of the amylin protofibril, a region that may contribute to protofibril stabilization and lateral association. Moreover, structurally related polyphenolic analogues containing a higher number of hydroxyl groups displayed stronger predicted binding affinities through cooperative hydrogen-bond networks involving Ser20 and Ser28, suggesting that polyphenolic enrichment may enhance anti-aggregating activity. These findings provide a structural rationale for the anti-amyloid effects of resveratrol and identify promising scaffolds for the future development of more potent amylin aggregation inhibitors. In agreement with this, our *in vivo* data indicate that the reduction in amyloid burden is associated with decreased activation of ER stress pathways, as evidenced by reduced phosphorylation of PERK and eIF2α. Given that ER stress is a key driver of β-cell apoptosis (25, 26), these results suggest that resveratrol may exert its protective effects primarily through modulation of proteotoxic stress responses in pancreatic tissue. Consistent with this interpretation, Ki67 immunostaining revealed no significant differences among the experimental groups, although HFD-fed TghIAPP mice tended to exhibit a lower proliferative response than HFD-fed Wt animals, while resveratrol treatment showed a tendency to increase Ki67 immunoreactivity. Together, these findings suggest that the beneficial effects of resveratrol are primarily associated with restoration of pancreatic tissue homeostasis rather than with a direct proliferative effect.

In addition to ER stress, mitochondrial dysfunction has been proposed as a contributor to β-cell failure in the context of amylin aggregation. Consistent with previous findings (5, 25), we observed alterations in mitochondrial dynamics in TghIAPP mice, characterized by increased p-DRP1 levels. Phosphorylation of DRP1 is one of the main mechanisms regulating its activity, promoting its recruitment to the mitochondrial outer membrane and facilitating mitochondrial fission. Under pathological conditions, increased p-DRP1 has been associated with excessive mitochondrial fragmentation, impaired mitochondrial function, and enhanced susceptibility to apoptosis, thereby contributing to disease progression. Therefore, the increased p-DRP1 levels observed in our model are consistent with a shift toward enhanced mitochondrial fission, which may contribute to β-cell dysfunction in the presence of amylin aggregates. However, resveratrol treatment did not significantly reverse these mitochondrial changes in the pancreas. This may reflect a more complex role of mitochondrial remodeling, potentially linked to adaptive responses such as mitophagy (27), and suggests that the primary effects of resveratrol in this model are more closely associated with ER stress modulation than with direct restoration of mitochondrial dynamics.

Beyond the pancreas, we explored the presence and potential consequences of amylin aggregation in the liver. TghIAPP mice, particularly under HFD conditions, exhibited increased hepatic lipid accumulation, inflammation, and apoptosis, accompanied by the presence of amyloid oligomers. These findings are consistent with emerging evidence suggesting that amylin aggregates may exert systemic effects and contribute to metabolic dysfunction beyond pancreatic tissue (28). Notably, although apoptosis was already increased in TghIAPP mice fed the standard diet, likely as a consequence of the presence of amylin oligomers, the additional metabolic stress induced by HFD further exacerbated lipid accumulation and inflammation, both of which are well-established drivers of apoptosis. This may explain why the anti-apoptotic effect of resveratrol was observed predominantly under HFD conditions, where its ability to reduce lipid accumulation and inflammation is expected to have a greater impact. However, the effects of resveratrol in the liver were more modest, with only partial or non-significant improvements in several parameters. While resveratrol reduced markers of inflammation and apoptosis, it did not significantly ameliorate ER stress or mTORC1 pathway activation. Similarly, Ki67 immunostaining showed only modest changes, with a significant increase observed exclusively in HFD-fed Wt mice when compared to STD-fed Wt mice treated with resveratrol, while no additional differences were detected among the remaining groups. These findings suggest that proliferative responses play a limited role in the hepatic phenotype under our experimental conditions. Moreover, although several hepatic parameters showed changes consistent with amylin-associated liver alterations, some outcomes exhibited only modest effects or trends, indicating that the hepatic phenotype is less pronounced than that observed in the pancreas. Therefore, these findings should be interpreted with caution and support the notion that the hepatic effects of amylin aggregation, as well as the therapeutic impact of resveratrol, are relatively limited under the experimental conditions evaluated.

The potential involvement of amylin aggregation in the brain, particularly in relation to neurodegenerative processes, has been increasingly recognized (8–12). In the present study, amyloid aggregates were detected in the hippocampus of TghIAPP mice, with a preferential distribution in specific subregions such as CA1 and the dentate gyrus. This regional specificity is consistent with previous reports highlighting differential vulnerability within the hippocampus (29, 30). However, the functional and mechanistic implications of these findings remain uncertain. Importantly, although amyloid aggregates were detected in the hippocampus, the present data do not establish a causal relationship between amylin deposition and neurological dysfunction. Therefore, the neuropathological significance of these findings should be interpreted with caution. Previous *in vitro* studies have demonstrated the beneficial effects of resveratrol on mitochondrial function in the hippocampus of mice with lipopolysaccharide-induced depressive behaviors (31), suggesting that, despite its limited *in vivo* efficacy, resveratrol can still improve specific mitochondrial parameters. However, its effects on amyloid burden and behavioral performance were limited, in particular, the observed improvements in behavioral parameters did not reach statistical significance and should therefore be interpreted with caution.

Several limitations of this study should be considered when interpreting the brain-related findings. The use of pooled hippocampal samples for biochemical analyses reduces the ability to assess biological variability and limits the strength of the conclusions drawn from these data. Moreover, the relatively modest amyloid burden observed in the hippocampus, together with the limited behavioral alterations and the modest effects of resveratrol in brain tissue, further support a cautious interpretation of these findings. Together, these factors indicate that the brain-related observations should be regarded as exploratory and warrant further investigation using approaches with higher resolution and statistical power. Although our findings demonstrate the presence of amylin aggregates in the hippocampus, they do not allow definitive conclusions regarding their contribution to neurological dysfunction or neurodegenerative processes. Additional studies employing functional and mechanistic approaches with higher resolution and statistical power will be required to establish the potential involvement of amylin aggregation in central nervous system pathology.

**Figure 7:**
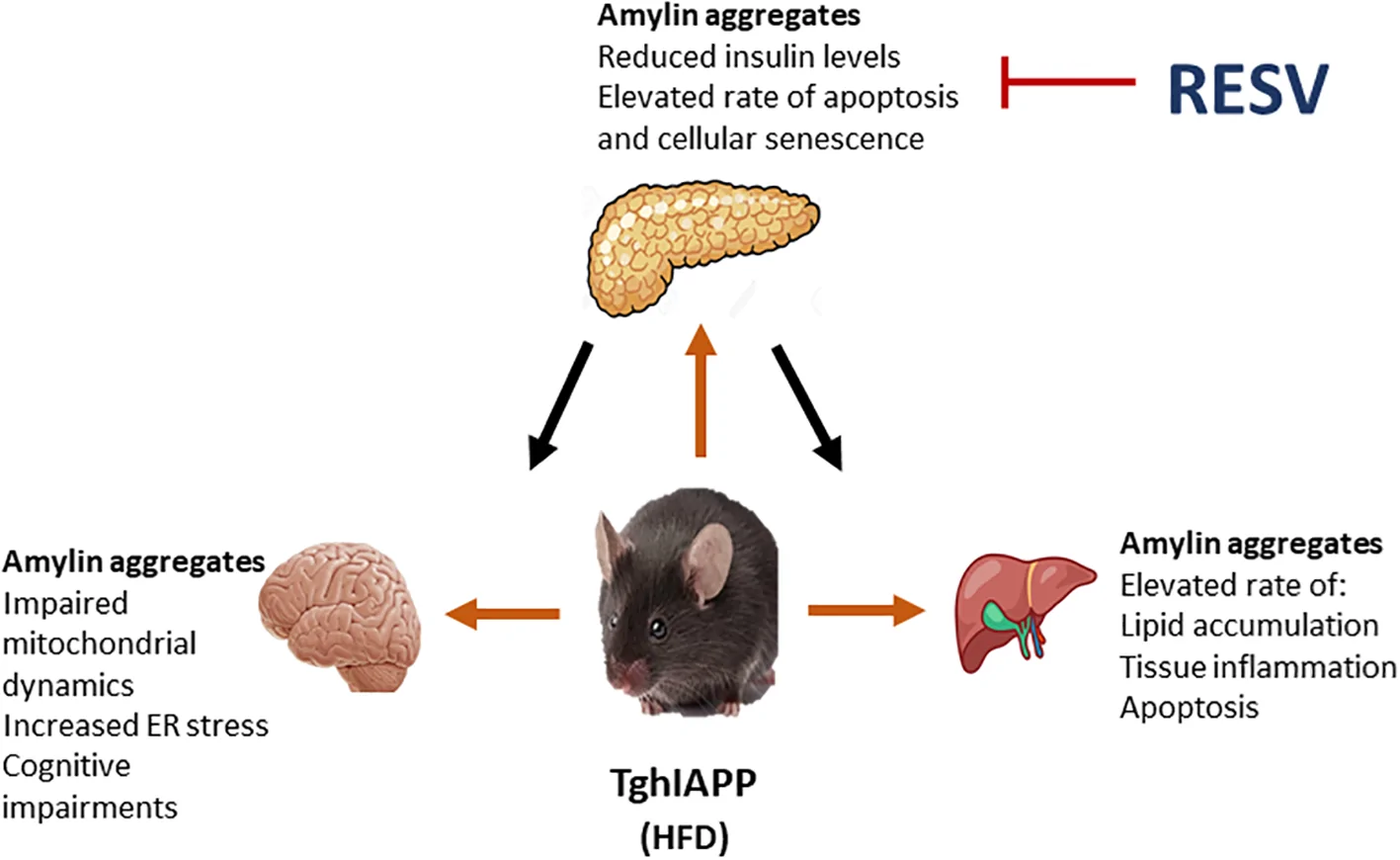
Schematic representation of pathological alterations in the TghIAPP mouse model. These transgenic mice express the human amylin gene specifically in the pancreas (Jackson Laboratory). When subjected to HFD, TghIAPP mice exhibit impaired glucose tolerance, accompanied by reduced insulin immunolabeling in pancreatic islets. These alterations are associated with increased apoptosis and cellular senescence, alongside dysregulation of critical signaling pathways within the pancreatic tissue. As a result of these changes, human amylin is distributed to peripheral organs, including the liver and brain, where it contributes to the disruption of tissue homeostasis. In the liver, its accumulation correlates with elevated inflammation and apoptosis. In the brain, the presence of amylin aggregates perturbs endoplasmic reticulum stress pathways and interferes with mitochondrial dynamics. Notably, treatment with resveratrol mitigates the pathological changes observed in the pancreas by significantly reducing amylin aggregate formation, apoptosis, and cellular senescence.

Collectively, our findings support a central role for amylin aggregation in pancreatic dysfunction and suggest that resveratrol can modulate key pathological processes in this tissue. In contrast, the effects observed in liver and brain are more variable and less pronounced, highlighting the tissue-specific nature of both amylin toxicity and therapeutic responses. These results underscore the importance of carefully distinguishing between primary and secondary effects when evaluating multi-organ involvement in complex metabolic diseases. It should be noted, however, that the present study was not designed to elucidate the molecular mechanisms underlying the effects of resveratrol. Rather, our primary objective was to evaluate its ability to reduce amylin aggregation and attenuate the associated tissue alterations. Therefore, the observed changes in ER stress- and mitochondrial dynamics-related markers should be interpreted as potential pathways associated with the protective effects of resveratrol, rather than as evidence of direct mechanistic actions. Future studies should aim to further elucidate the mechanisms underlying the systemic distribution of amylin aggregates, including the potential role of extracellular vesicles in mediating inter-organ communication (6). Further studies specifically addressing autophagic flux, mitochondrial function, oxidative stress, and other signaling pathways will also be required to define the molecular mechanisms responsible for the protective effects of resveratrol. In addition, strategies to improve the bioavailability and tissue penetration of resveratrol (32) may enhance its therapeutic potential, particularly in the central nervous system. The main conclusions derived from the present study are resumed in **Figure 7**.

### 4.1 Study limitations

The present study has some limitations that should be considered when interpreting the findings. The number of animals was not identical across all experimental groups. This variation reflected the availability of transgenic animals during the breeding program, which limited the generation of perfectly balanced experimental groups. A sample size estimation performed for the present experimental design indicated that the sample sizes included in the study generally met, or closely approached, the estimated number of animals required to achieve the desired statistical power. Therefore, although unlikely to have influenced the overall conclusions of the study, the unequal group sizes should be taken into consideration when interpreting the results.

## Data Availability

The original contributions presented in the study are included in the article/**Supplementary Material**. Further inquiries can be directed to the corresponding author.

## Supplementary material

Supplementary Figure 1 Insulin tolerance test (ITT) curves were generated for all experimental groups of mice. All data are presented as mean ± standard error of the mean (SEM), *n* = 5–10.

Supplementary Figure 2 Representative immunohistochemistry images of ki67 staining in pancreatic tissue, with corresponding quantification **(A, B)** and in liver tissue **(C, D)**. All data are presented as mean ± standard error of the mean (Wt STD Vehicle: n = 5; Wt STD RESV: n = 6; Wt HFD Vehicle: n = 7; Wt HFD RESV: n = 7; Tg STD Vehicle: n = 8; Tg STD RESV: n = 8; Tg HFD Vehicle: n = 7; Tg HFD RESV: n = 10). A two-way ANOVA was performed, with statistical significance defined as follows: *p < 0.05; **p < 0.01; ***p < 0.001; ****p < 0.0001.

Supplementary Figure 3 Representative immunofluorescence images of the entire hippocampus, with amyloid aggregates labeled in green using Thioflavin T and nuclei counterstained in blue with DAPI. All data are presented as mean ± standard error of the mean (Wt STD Vehicle: n = 5; Wt STD RESV: n = 6; Wt HFD Vehicle: n = 7; Wt HFD RESV: n = 7; Tg STD Vehicle: n = 8; Tg STD RESV: n = 8; Tg HFD Vehicle: n = 7; Tg HFD RESV: n = 10). A two-way ANOVA was performed, with statistical significance defined as follows: *p < 0.05; **p < 0.01; ***p < 0.001; ****p < 0.0001.

Supplementary Figure 4 Representative immunofluorescence images of the prefrontal cortex, showing amylin aggregates labeled in green with Thioflavin T and nuclei counterstained in blue with DAPI. All data are presented as mean ± standard error of the mean (Wt STD Vehicle: n = 5; Wt STD RESV: n = 6; Wt HFD Vehicle: n = 7; Wt HFD RESV: n = 7; Tg STD Vehicle: n = 8; Tg STD RESV: n = 8; Tg HFD Vehicle: n = 7; Tg HFD RESV: n = 10). A two-way ANOVA was performed, with statistical significance defined as follows: *p < 0.05; **p < 0.01; ***p < 0.001; ****p < 0.0001.

Supplementary Figure 5 Western blot analysis was performed on prefrontal cortex tissue. Due to sample limitations, cortices from multiple mice within each experimental group were pooled (*n* = 5–10) for analysis. **(A)** Protein levels of p-ULK1-757/ULK1; **(B)** p-PERK/PERK and p-eIF2α/eIF2α; and **(C)** p-DRP1/DRP1, Mfn1, and Mfn2 were assessed. All data are presented as the mean ± standard error of the mean (SEM).

Supplementary Figure 6 Chemical structures of resveratrol (Molport-002-499-801) and the 22 structurally related analogues retrieved from the MolPort database. The three compounds which showed the most favorable predicted binding energies are highlighted in red.

Supplementary Table 1 Predicted binding energies of the selected docking pose for each ligand obtained with AutoDock4.2.
